# Cooperative DNA binding by proteins through DNA shape complementarity

**DOI:** 10.1093/nar/gkz642

**Published:** 2019-07-25

**Authors:** Stephen P Hancock, Duilio Cascio, Reid C Johnson

**Affiliations:** 1 Department of Biological Chemistry, David Geffen School of Medicine at the University of California at Los Angeles, Los Angeles, CA 90095-1737, USA; 2 Department of Chemistry, Towson University, 8000 York Rd., Towson, MD 21252, USA; 3 University of California at Los Angeles-Department of Energy Institute of Genomics and Proteomics, University of California at Los Angeles, Los Angeles, CA 90095-1570, USA; 4 Molecular Biology Institute, University of California at Los Angeles, Los Angeles, CA 90095, USA

## Abstract

Localized arrays of proteins cooperatively assemble onto chromosomes to control DNA activity in many contexts. Binding cooperativity is often mediated by specific protein–protein interactions, but cooperativity through DNA structure is becoming increasingly recognized as an additional mechanism. During the site-specific DNA recombination reaction that excises phage λ from the chromosome, the bacterial DNA architectural protein Fis recruits multiple λ-encoded Xis proteins to the *attR* recombination site. Here, we report X-ray crystal structures of DNA complexes containing Fis + Xis, which show little, if any, contacts between the two proteins. Comparisons with structures of DNA complexes containing only Fis or Xis, together with mutant protein and DNA binding studies, support a mechanism for cooperative protein binding solely by DNA allostery. Fis binding both molds the minor groove to potentiate insertion of the Xis β-hairpin wing motif and bends the DNA to facilitate Xis-DNA contacts within the major groove. The Fis-structured minor groove shape that is optimized for Xis binding requires a precisely positioned pyrimidine-purine base-pair step, whose location has been shown to modulate minor groove widths in Fis-bound complexes to different DNA targets.

## INTRODUCTION

Cooperative interactions between DNA-binding proteins at specific genomic sites govern many cellular processes including transcription, replication, and recombination. Whereas individual affinities and sequence selectivity of participating proteins in these reactions can be remarkably poor, favorable interactions between binding partners can synergistically promote assembly of multi-component nucleoprotein complexes and underlie mechanisms of combinatorial control.

Most reported instances of binding cooperativity on DNA involve direct protein–protein interactions, but there are examples whereby local protein-induced changes in DNA structure have been implicated in promoting binding of partner proteins. By this mechanism, binding of one protein to a specific site changes DNA shape so as to create an optimized DNA conformation for a second protein that would otherwise exhibit poorer binding affinity to that site. An often cited example is the cooperative binding of eight different transcription factors within a 55 bp segment of the interferon-β enhanceosome, where X-ray crystallography revealed few direct protein–protein interactions between binding partners (1). Evidence has also been presented indicating that conformational features of DNA can be transmitted over distances of one or two helical turns to influence binding kinetics of different protein pairs (2).

**Figure 1.**
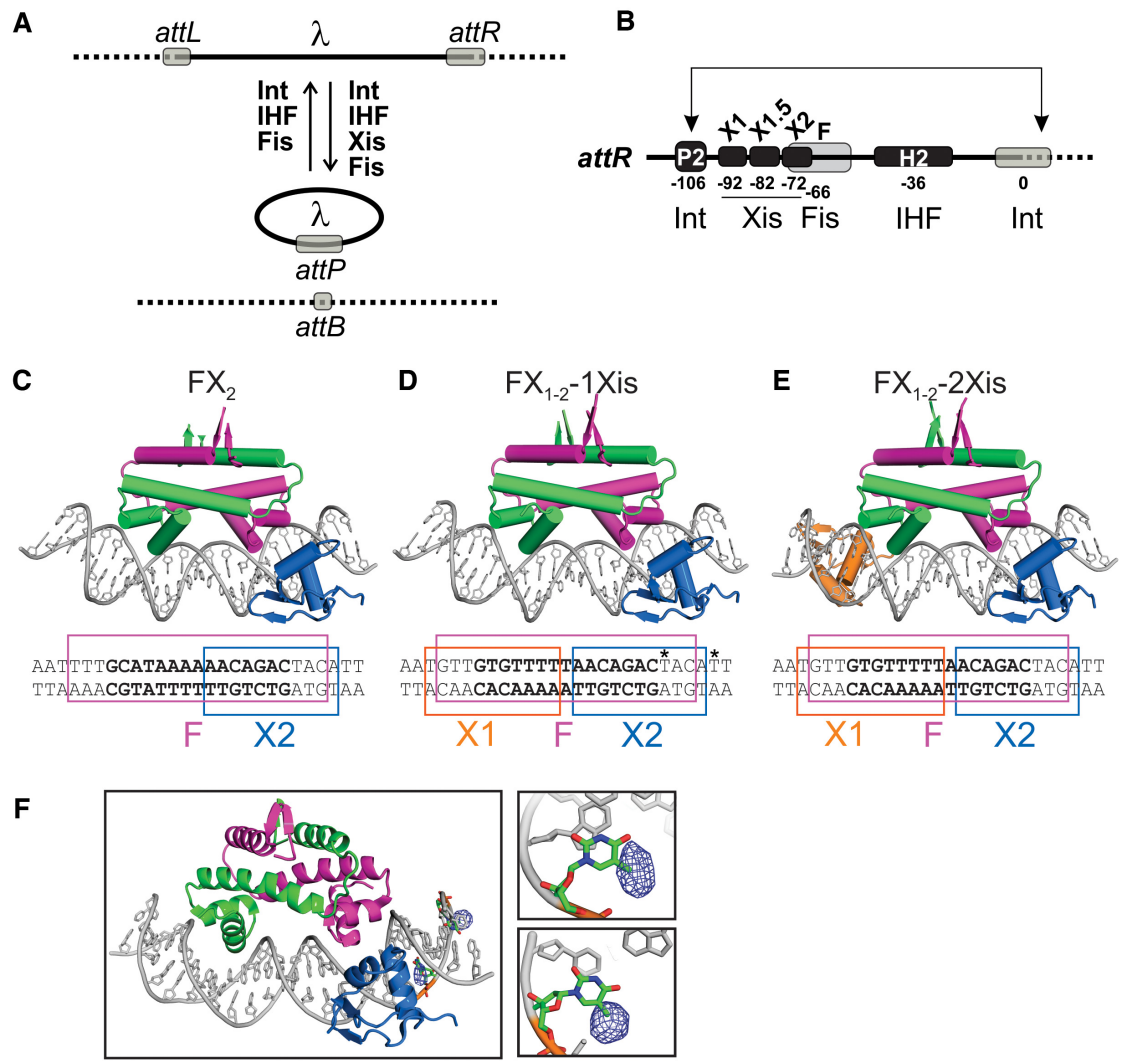
The phage λ excision reaction and Fis–Xis–DNA structures. (**A**) λ excision and integration reactions. The recombination reaction excising the phage genome (thick line) from the *E. coli* chromosome (dash line) occurs between specific recombination sites *attL* and *attR* and requires phage-encoded integrase (Int) and Xis plus bacterial nucleoid proteins IHF and Fis. The reverse reaction, integrative recombination, requires Int and IHF and is also stimulated by Fis but inhibited by Xis (3). (**B**) Organization of the *attR* recombination site with proteins and binding sites denoted. In the *attR* x *attL* recombination complex the bivalent Int recombinase simultaneously binds to the P2 and the bacterial DNA side of the crossover region (0) (12). The combined DNA bending activities of Xis, Fis and IHF stereo-specifically loop the DNA to enable formation of this bridge by the Int N- and C-terminal domains. (**C–E**) X-ray structures of three Fis–Xis–DNA ternary complexes: FX_2_, FX_1-2_-1Xis and FX_1-2_-2Xis, respectively. The DNA sequences of the oligonucleotides used for crystallography are given below with the boundaries of the Fis and Xis binding sites denoted; the Fis core sequence is in bold letters. Fis dimers (magenta and green subunits) bind DNA (grey) in a similar manner to that observed in Fis-DNA binary complexes. The Xis^1–55^ bound to the X2 (blue) site inserts its helix B into the major groove and its β-turn-β wing motif into the minor groove towards the center of the Fis binding interface. Xis^1–55^ bound within the X1 region (orange) in the FX_1-2_-2Xis structure inserts its wing motif into the minor groove at the outer edge of the Fis binding site and thus is in the reverse orientation. (**F**) FX_1-2_ DNA substituted with 5-bromodeoxyuridine (5-BrdU) at the sequence positions denoted with asterisks in panel D was used to verify the site of Xis^1–55^ binding in the FX_1-2_-1Xis structure. Difference density (blue mesh) of the 5-BrdU is rendered at 5σ. The right panels highlight nts 8 (top) and 12 (bottom) (numbered from the central T) that are substituted with 5-BrdU.

A well-studied example of cooperative binding interactions involving DNA architectural and recombinase proteins are the mutually exclusive assembly of recombination complexes that control the integration and excision reactions of the phage λ genome (Figure 1A) (3). Multiple copies of four different proteins binding over ∼250 bp of phage and bacterial DNA participate in these reactions. In this paper, we focus on the ability of the *Escherichia coli* Fis protein to recruit the phage-encoded Xis protein to the *attR* recombination site, a key control step in formation of the excisive intasome (Figure 1B) (4,5). Fis binds to the high affinity site F located ∼ 65 bp from the region that undergoes DNA strand exchange by the phage integrase. Binding of the Fis dimer recruits an Xis protomer to the X2 site that overlaps F, which leads to sequential binding of two additional Xis protomers to the adjacent DNA segment (sites X1.5 and X1) (6–8). Xis binding at X1 is critical because it recruits the N-terminal domain of the λ integrase protein (Int) to the adjacent P2 site, which is uniquely occupied in the excisive intasome (9–11). The curvature of DNA induced by the Fis-3Xis nucleoprotein filament, together with bending by the architectural protein IHF bound at the H2 site, positions the C-terminal catalytic domain of the P2-bound Int molecule at the DNA crossover region (Figure 1B) (12).

Fis is a bacterial nucleoid protein that functions in transcription, replication, and recombination reactions (13). Fis can stably bind to specific DNA sites, although the sequences of these high affinity binding sites vary considerably. The Fis dimer binds DNA through its helix-turn-helix motifs, and multiple co-crystal structures have shown that the DNA in Fis complexes is highly distorted, exhibiting both radically changing minor groove widths and significant overall curvature (60–75°) (14–16).

Xis is a winged-helix DNA binding protein, which is expressed at high levels shortly after prophage induction, but by itself, exhibits low binding specificity (8,10,17,18). Indeed, addition of the X2 binding sequence to a DNA fragment does not enhance Xis binding *in vitro*, but Xis cooperatively assembles a 3-Xis protomer complex on *attR* DNA containing the native X2–X1.5–X1 binding sites (6–8). The Xis nucleoprotein filament structure assembles at lower Xis concentrations in the presence of Fis (7,8,18–20). Moreover, without Fis *in vivo, attL* × *attR* recombinant products are reduced ∼100-fold from an induced λ lysogen, which results in up to a 1000-fold decrease in phage yields (4,8).

Previous work mapped the overlapping F and X2 binding sites on *attR* but revealed surprisingly small effects by Fis and Xis residues on Fis–Xis binding cooperativity (8,19). These data, along with subsequent X-ray structures of Fis bound to DNA revealing large DNA conformational changes (14–16), led to the suggestion that DNA structural changes could be playing a prominent role in the targeting of Xis to X2 by Fis. We investigate this idea by solving three different crystal structures of Fis–Xis–DNA ternary complexes and compare these to previously determined binary structures containing DNA bound by only Fis or Xis. These structures, together with accompanying mutagenesis and biochemistry, show that direct protein–protein interactions cannot account for Fis–Xis cooperativity. Rather, we posit that Fis-induced changes in DNA shape target Xis binding to the *attR* locus.

## MATERIALS AND METHODS

### Crystallization, structure determination and refinement

Fis (16) and Xis (8,18) proteins were prepared as previously described. Xis^1–55^ containing residues 1–55 with a cysteine to serine substitution at residue 28 was used for crystallography. Xis mutations were introduced using the QuikChange method into the codon-optimized full-length Cys28Ser *xis* gene cloned between the NdeI and BamHI sites of pET11a (pRJ2178). DNA oligonucleotides for crystallography and *in vitro* binding assays were obtained from IDT and annealed in equal molar amounts in buffer containing 20 mM Tris–HCl (pH 8.0) and 150 mM sodium acetate. The DNA sequences used for each structure are given in Figure 1C–E.

Fis–Xis–DNA complexes were prepared for crystallization by first incubating Fis with DNA in crystallization buffer containing 20 mM Tris–HCl (pH 7.5) and 200 mM Na acetate with 1.25 molar excess of Fis dimer to DNA duplex at a protein concentration of 0.5 mg/ml. After 20 min at 4°C, Xis^1–55^ was added at 2- or 4-fold molar excess relative to Fis. Following an additional 30 min incubation, the complex was concentrated to ∼10 mg/ml using a 3K MWCO centricon filter (Amicon). Optimal crystals were grown at 4°C in hanging drops containing equal volumes of complex solution and reservoir solution (0.1 M HEPES, pH 7–7.5 and 15% (v/v) PEG4000) and began to appear after 1-2 weeks of growth. Crystals were cryoprotected in reservoir solution plus 30% PEG4000, and diffraction data were collected at the Advanced Photon Source, Chicago IL, beamline 24-ID-C.

Diffraction data were indexed and scaled with XDS and XSCALE (21). The FX_1-2_ crystals were fragile, sensitive to radiation damage, and exhibited anisotropic diffraction. The FX_1-2_-1Xis structure was the first to be solved by molecular replacement (MR) with PHASER (22) using the Fis-DNA binary complex (PDB code: 3IV5) as the search model. Rigid body fit of the Xis monomer (PDB code: 1RH6, chain B) into the positive difference density was performed manually in COOT (23). Models were refined in PHENIX (24) and BUSTER (25), and further model building and validation was performed using COOT. The FX_1-2_-1Xis structure was refined at 3.60 Å resolution generating final *R*/*R*_free_ values of .199/.248 with an average B of 122 Å^2^. Given the modest resolution of the FX_1-2_-1Xis structure, the Xis-bound half-site was unambiguously identified in a different crystal of this complex by anomalous dispersion from two asymmetrically positioned bromine atoms incorporated as 5-bromodeoxyuridine base analogs within the Xis bound half-site (see Figure 1D and F). The FX_1-2_-2Xis and the higher resolution FX_2_ structures were solved in a similar manner using FX_1-2_-1Xis as the MR search model. Positive difference maps clearly showed the presence of a second Xis monomer in the asymmetric unit of the FX_1-2_-2Xis structure, which was again manually fit as a rigid body in COOT. FX_1-2_-2Xis was refined at 3.30 Å to an *R*/*R*_free_ of .192/.219 (average *B* = 134 Å^2^), and FX_2_ was refined at 2.7 Å to *R*/*R*_free_ values of .222/.227 with an average B value of 73 Å^2^ but lower *B* values over the region co-bound by Xis where many solvent molecules (average *B* = 53 Å^2^) are resolved. Table 1 provides diffraction and refinement statistics for the three structures. DNA structural parameters were calculated using 3DNA (26), and DNA curvature determined using CURVES (27). Shape complementarity (28) and surface area (areaimol; (29)) calculations were obtained using the CCP4 suite of crystallography programs (30). Models and structure figures were generated using PyMol (Schrödinger, http://www.pymol.org).

**Table 1.** X-ray diffraction data and refinement statistics

	FX_2_	FX_1-2_-1Xis	FX_1-2_-2Xis
PDB code	6P0S	6P0T	6P0U
Data collection			
Beamline	APS 24 ID-C	APS 24 ID-C	APS 24 ID-C
Space group	*P*4_3_2_1_2	*P*4_3_2_1_2	*P*4_3_2_1_2
Unit cell dimensions			
*a, b, c* (Å)	107.7, 107.7, 150.4	108.2, 108.2, 152.6	151.9, 151.9, 120.2
α, β, γ (°)	90.0, 90.0, 90.0	90.0, 90.0, 90.0	90.0, 90.0, 90.0
Wavelength (Å)	0.9717	0.9793	0.9795
Resolution range (Å)^a^	87.6–2.7 (2.8–2.70)	88.3–3.6 (3.7–3.6)	80.2–3.3 (3.4–3.3)
Measured reflections	222 119 (10 039)	140 007 (9561)	169 400 (12 396)
Unique reflections	23 763 (1528)	10 977 (753)	20 814 (1491)
*R*_merge_^b^	11.6 (49.2)	11.8 (81.6)	10.7 (81.9)
CC_1/2_	99.8 (91.5)	99.9 (84.8)	99.9 (87.1)
*I*/σ(*I*)	12.5 (3.7)	13.1 (3.1)	15.14 (2.24)
Completeness (%)	95.6 (85.1)	99.8 (97.2)	96.0 (96.1)
Refinement			
Resolution (Å)	2.7	3.6	3.3
No. of reflections	23762	10974	20813
*R*_work_	22.2	19.9	19.2
*R*_free_^c^	23.0	24.8	21.9
RMSD bond length (Å)	0.01	0.004	0.010
RMSD bond angle (°)	1.0	0.64	1.05
No. of atoms			
Protein	1727	1699	2047
Nucleic acid	1101	1101	1101
Water	58	0	0
*B* factors			
Protein	72.1	118.9	134.3
DNA	75.9	127.4	133.5
Solvent	53.2		
Ramachandran statistics			
Favored	91.5	92.7	91.1
Allowed	8.5	7.3	8.9
Generously allowed	0	0	0

^a^Values in parentheses refer to the highest resolution shell.

^b^ *R*_merge_= Σ | *I* – <*I*> | / Σ *I*.

^c^Calculated using 4.8–5.1% of the data.

### *In vitro* binding assays

*In vitro* protein-DNA complexes containing Fis and/or Xis were analyzed by electrophoretic mobility shift assays as previously described (8) with slight modifications. For ternary complexes on 34 bp DNA duplexes, 50–200 fmol of 5′ ^32^P labeled DNA (*attR* coordinates –54 to –87) was incubated with 4 nM Fis in binding buffer (20 mM HEPES (pH 7.5), 0.1 M NaCl, 50 μg/ml poly dI/dC competitor DNA, 5% (v/v) glycerol, 500 μg/ml BSA, 1 mM EDTA and 1 mM DTT) for 20 min. Xis was then added (0.05–1.35 μM) and incubated for 30 min at 25°C in a final volume of 20 μl. Complexes were then separated from free DNA by electrophoresis in 8%, 37.5:1 acrylamide/bis-acrylamide gels in 45 mM Tris-borate EDTA buffer and quantified on a Typhoon 9400 phosphorimager (GE Life Sciences). Equilibrium dissociation constants were estimated from a plot of the ratio of the Fis–Xis-bound DNA signal to the Fis-bound DNA signal as a function of log Xis concentration as adapted from (31).

**Figure 2.**
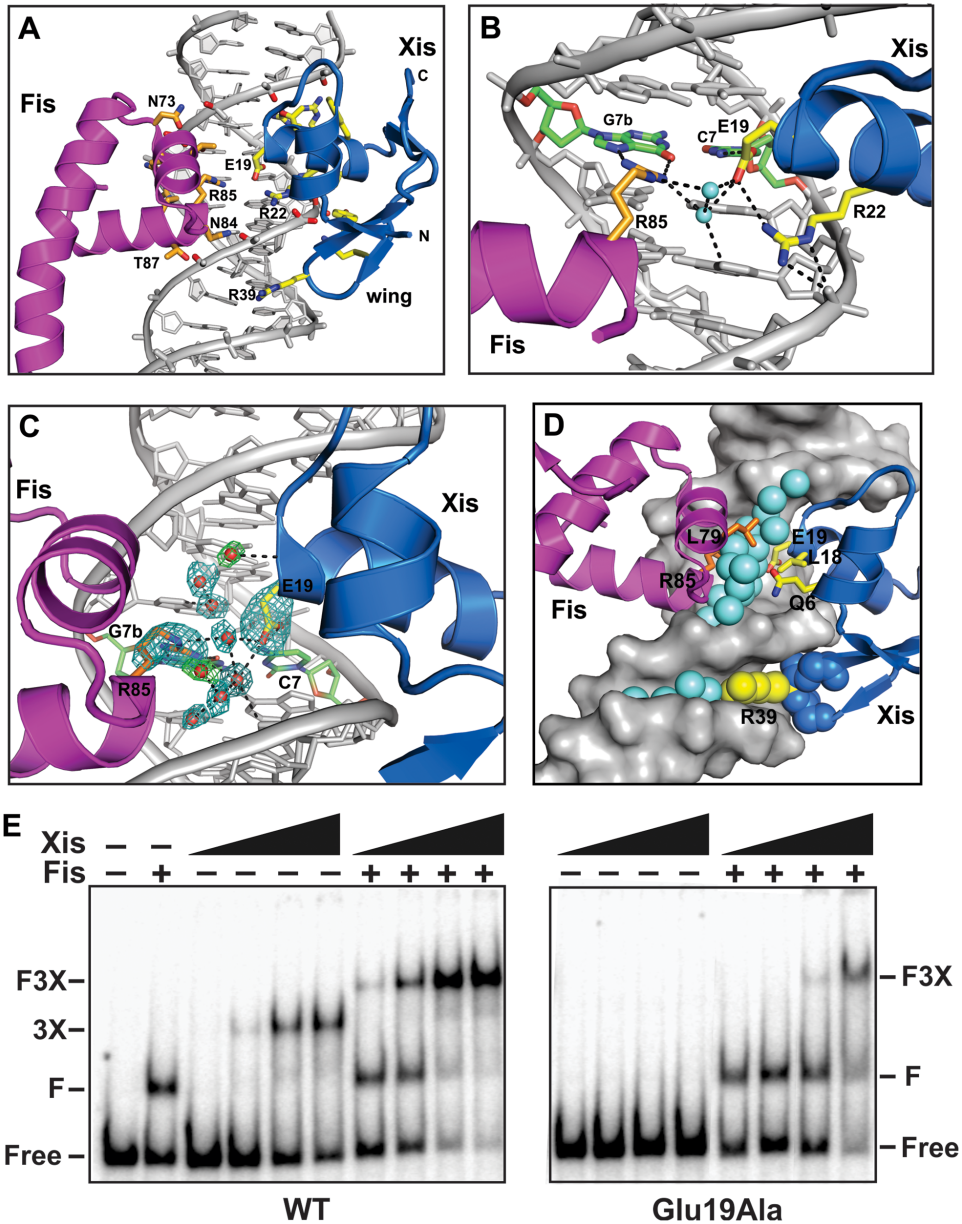
Molecular interactions between Fis, Xis, and DNA. (**A**) The FX_2_ structure over the X2 binding site. The side chains of Fis (orange) and Xis (yellow) residues that contact DNA along with DNA phosphate oxygen atoms (red) are highlighted. Only helices B–D of subunit A of the Fis dimer is displayed. (**B**) The DNA major groove interface with Fis and Xis. The recognition helices of Fis and Xis along with Fis Arg85, Xis Glu19 and Arg22, and two interconnected water molecules (cyan spheres) are displayed. Dashed lines represent hydrogen bonding. Fis Arg85 and Xis Glu19 most closely approach each other within the major groove and are directly hydrogen bonded to the same C7/G7b base pair. (**C**) *F*_o_ – *F*_c_ difference omit map showing a subset of interfacial water molecules between Fis and Xis within the major groove, along with the Fis Arg85 and Xis Glu19 side chains (omit density contoured at 5.0σ). Omit maps for the solvent molecules were individually generated and contoured at 4.5–5.0σ (blue) or 3.25σ (green). The C7/G7b base pair is colored. (**D**) View of the Fis–Xis binding region highlighting the layer of solvent molecules (cyan spheres) separating Fis and Xis residues in the major groove. Xis backbone atoms within the wing (Gly38–Glu40, blue) along with Arg39 side chain atoms (yellow) that insert into the minor groove are rendered as spheres; ordered waters within the minor groove also displayed. (**E**) Gel mobility shift assays evaluating DNA binding by Xis-wt and Glu19Ala on an *attR* DNA fragment with and without Fis. Xis concentrations increased from 50 to 1350 nM in 3-fold increments and Fis was added at 4 nM as designated. Xis-wt cooperatively binds to *attR* to forms a complex containing three Xis protomers (3X). Xis-wt binding is stimulated in the presence of Fis to assemble a Fis dimer + 3Xis – *attR* complex (F3X). DNA complexes by Xis-Glu19Ala alone are not detectable, but F3X complexes are formed at high mutant Xis concentrations demonstrating cooperative binding with Fis without the Glu19 side chain.

Complex formation was also measured on a 263 bp fragment amplified from the λ prophage *attR* (*attR* coordinates –220 to +43) using 5′ ^32^P-labeled primers as described in Papagiannis *et al.* (8). Binding buffer contained 5.0 μg/ml poly dI/dC (Figure 2) or 12.5 μg/ml sonicated salmon sperm DNA (Figure 6) and ∼200 fmol of probe DNA. Following a 20 min incubation at 25°C with Fis, Xis was added in increasing concentrations as specified in the figure legends, and the reactions were equilibrated for an additional 30 min. Binding reactions were subjected to native PAGE in 6% 37.5:1 acrylamide/bis-acrylamide gels, which were then dried and imaged.

## RESULTS

### X-ray crystal structures of three Fis–Xis–DNA ternary complexes

To explore the structural basis for Fis recruitment of Xis to the X2 site within the λ prophage *attR* recombination site, X-ray crystal structures of three different Fis–Xis–DNA complexes were determined (Table 1). The ‘FX_2_’ structure contains a Fis dimer, an Xis^1–55^ monomer (hereafter referred to as Xis), and a 27 bp DNA duplex representing the native sequence except for an A/T base pair at each end (Figure 1C). The 27 bp DNA used for the ‘FX_1-2_-1Xis’ and ‘FX_1-2_-2Xis’ crystals contains a Fis binding site with overlapping X1 and X2 sequences on either side. The ‘FX_1-2_-1Xis’ structure contains a Fis dimer plus Xis bound at X2 (Figure 1D). We verified that the single Xis protomer binds to the X2 site in the X_1-2_F-1Xis structure by incorporating 5-bromo-deoxyuracil (5-BrdU) in place of deoxythymidine bases that are asymmetrically distributed within the X_1-2_ DNA (Figure 1D sequence). Anomalous dispersion from Br was used to calculate an anomalous difference map to locate the Br atoms in the model-phased ternary structure; the difference density confirms that Xis is bound to the X2 half-site containing the brominated bases (Figure 1F). The ‘FX_1-2_-2Xis’ contains a second Xis protomer bound over the X1 end (Figure 1E). However, this Xis is bound in the reverse orientation with respect to the X1 binding site (6) and Fis (8). Although the structure of the Xis bound over X1 in the FX_1-2_-2Xis complex is not relevant to Fis–Xis cooperativity, we discuss below how it informs on Xis–Xis interactions leading to formation of the Xis nucleoprotein filament and why Xis bound to the X1 sequence in the correct configuration was not obtained.

**Figure 3.**
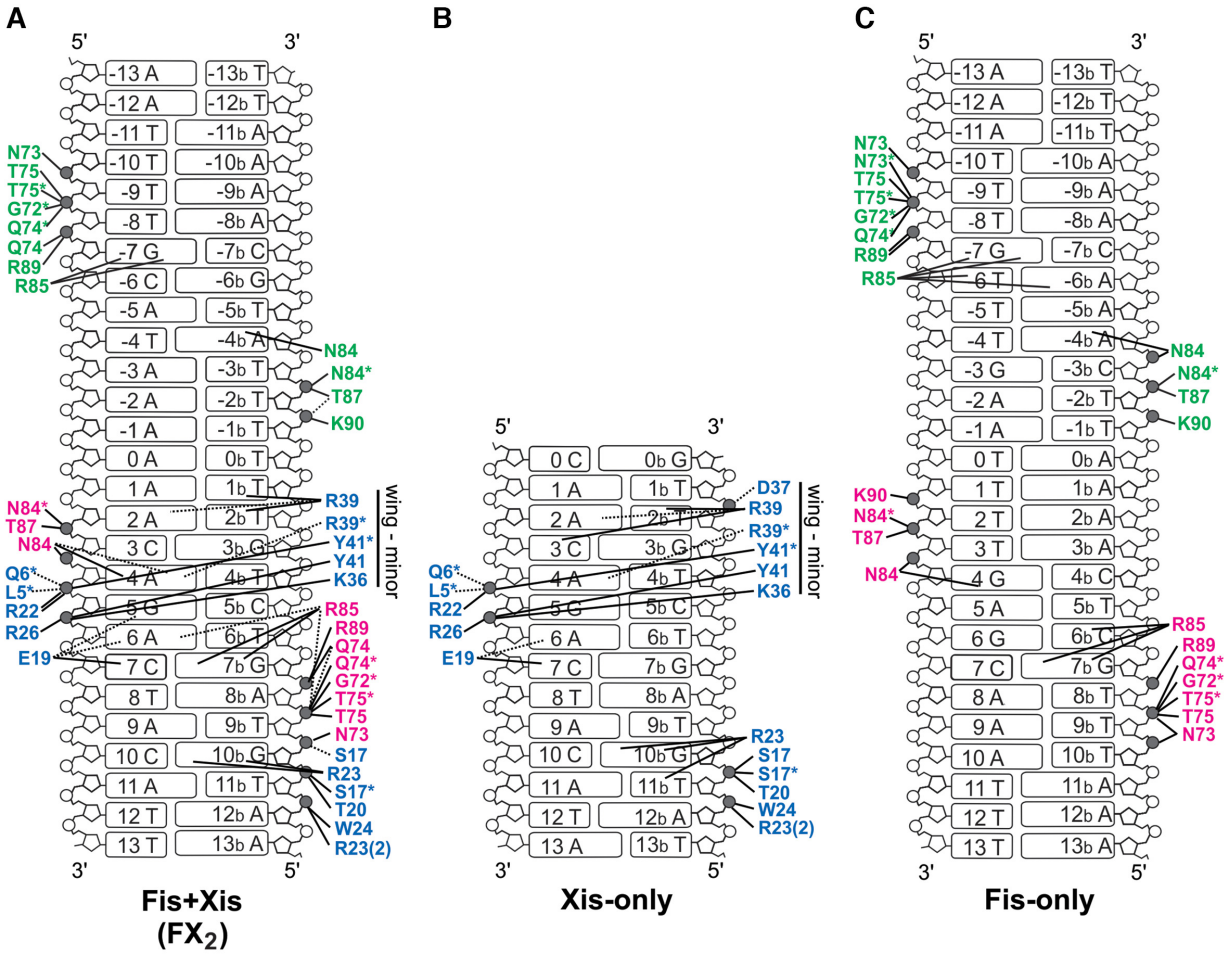
Contact diagrams of DNA complexes: (**A**) Fis + Xis (FX_2_), (**B**) Xis-X2 (derived from PDB codes 1RH6 (1.7 Å resolution) and 2OG0 (1.9 Å) with both rotamers of Arg23 depicted as found in 2IEF, 2.6 Å), and (**C**) Fis-F1 (PDB code 3IV5, 2.9 Å). Phosphates that are contacted directly by protein moieties are grey. Contacts made by Fis chain A (magenta) and chain B (green) and Xis (blue) residues are shown. Asterisks represent contacts made by protein backbone atoms. Direct hydrogen bonds are denoted by solid lines and water-bridged contacts by dashed lines.

The Xis winged-helix motif protein is bound to the X2 site in the FX_2_, FX_1-2_-2Xis and FX_1-2_-2Xis structures essentially identically when compared to each other and to Xis-X2 crystal structures (PDB codes 1RH6, 2OG0, and 2IEF; Figures 1C–E, 2A and 3). The DNA recognition α-helix of Xis in the Fis–Xis complexes is inserted into the major groove adjacent to but in an antiparallel orientation with respect to the recognition α-helix of the helix-turn-helix motif of Fis. The N-terminal ends of the Fis and Xis recognition helices that protrude into the major groove are rotationally shifted ∼70° from each other. As discussed further below, there is strikingly little direct contact between Xis and Fis residues within the major groove (Figure 2A–D). The Xis wing (β3-hairpin-β4) reaches over the DNA backbone and inserts into the minor groove towards the center of the Fis dimer binding site. The Arg39 side chain emanating from the hairpin tip travels along the floor of the minor groove making many nonspecific contacts with the DNA in a similar manner as observed in Xis-only DNA structures, but no Xis residues within the wing are close to Fis (Figure 2A and D). The Fis dimer structures in the Fis–Xis complexes are essentially identical to those in Fis or Fis-DNA structures; however, the N-terminal β-hairpin arms are not resolved in these crystals, as is the case with the dominant form of Fis-only crystals (32,33). We discuss molecular interactions within the Fis–Xis complex primarily using the higher resolution FX_2_ structure but note relevant differences present in the FX_1-2_-1Xis and FX_1-2_-2Xis structures.

### DNA contacts within the Fis–Xis–DNA ternary complex

Fis and Xis contacts to DNA in the ternary complexes are nearly identical to those observed in the previously reported binary complexes of Fis-DNA and Xis-DNA (Figures 2A and 3 (6,8,15–17). As is the case in the binary complexes, most of the protein contacts are to DNA phosphate backbone atoms. The most important direct base contact by Xis within the major groove is by Glu19, which is directly hydrogen bonded to C7:N4 and is involved in water-bridged contacts with A6 and G5 (Figure 2B and C). As found in other Xis-DNA complexes, Arg22 is bonded to Glu19 to help neutralize the charge and to the phosphate of G5. Arg23 has been found to adopt two conformations in different Xis-DNA structures: one in which it makes hydrogen bonds to bases (G10b and T9b) and a second in which it contacts the T11b phosphate. The Arg23 side chain is present in both conformations in the FX_2_ complex with the dominant rotamer contacting the DNA backbone, as is the case for the X2 site within the X2–X1.5–X1 microfilament structure.

The most important base-specific contact by Fis in high affinity Fis binding sites is mediated by the guanidinium group of Arg85 to a guanine, and in the FX_2_ structure, both the N7 and O6 atoms of G7b are within 3 Å (Figures 2B and 3). Thus, each base of the position 7 C/G pair is directly contacted by either Fis or Xis (Glu19-C7:N4, 2.7 Å). In addition, the Fis Asn84 side chain is positioned to make a long range hydrogen bond with A4:N7, as is typically seen when a purine is at that location in Fis binding sites (16).

The FX_2_ structure reveals for the first time a layer of interconnected solvent molecules spanning most of the major groove interface with Fis (Supplementary Figure S1). A few waters are involved in bridging interactions between bases and Fis side chains (e.g., Asn84 and Arg85). The majority are separately hydrogen bonded to either bases or Fis. These, together with additional interconnected stable waters, form a near contiguous solvent surface between Fis and DNA to help stabilize the complex. However, it appears unlikely that water-mediated interactions provide much sequence specificity from the chemical interactions revealed here and the body of knowledge regarding Fis-DNA interactions (13,15,16,34–38).

### Lack of direct contacts between Xis and Fis

Xis and the helix-turn-helix motif of Fis subunit A approach each other within the shared major groove, but a layer of ordered solvent molecules separates the two proteins (Figure 2C and D). Near the floor of the groove, the side chains of Fis Arg85 and Xis Glu19 that are hydrogen bonded to the position 7 (C/G) base pair most closely approach each other (4.0 Å in FX_2_ to 4.3 Å in FX_1-2_-2Xis). These two side chains are indirectly hydrogen bonded to each other through two intermediary water molecules (Figure 2B and C). Previous mutant studies have shown that Fis Arg85 is essential for binding DNA, including at the F site of *attR* (38–41). Xis-Glu19Ala binds DNA poorly and nonspecifically (42). To evaluate if a long range or water-bridged interaction between Xis Glu19 and Fis Arg85 is important for Xis-Fis binding cooperativity, we asked whether Xis-Glu19Ala could be recruited to Fis-bound *attR*. In the presence of competitor DNA, no binding by Xis-Glu19Ala to *attR* is detectable (Figure 2E). However, Fis + Xis-Glu19Ala complexes are formed, albeit less efficiently than with Xis-wt. These results confirm the importance of Glu19 for Xis-DNA binding, but more importantly, show that Fis is able to recruit Xis in the absence of the Xis Glu19 side chain. These results lead us to conclude that direct or indirect interactions between Fis Arg85 and Xis Glu19 are not essential for binding cooperativity.

Outside of the major groove, Xis Gln6 and Leu18 are positioned 3.5–7.3 and 4.5–4.9 Å from Fis Leu79, respectively, in the different structures (Figure 2D for FX_2_). Previous data showed that a glutamic acid substitution of Xis Gln6 exhibited a small reduction in Fis–Xis cooperative binding, but a Leu18Ala mutation had no demonstrable effect (8). The orientation of the Xis Gln6 side chain is variable in the different Fis–Xis–DNA structures; in the FX_2_ complex it approaches within 3.5 Å of Leu79 on Fis, but in the FX_1-2_-2Xis complex the electron density is consistent with the Gln6 side chain oriented away from Leu79 and in position to hydrogen bond with a DNA phosphate. Although Xis-Gln6Glu exhibited poorer DNA binding (8), the variable positions of the Gln6 side chain relative to Fis Leu79 in the X-ray structures suggest that an interaction between these residues is unlikely to be playing a significant role in cooperative binding.

### DNA structure in the Fis–Xis–DNA ternary complex

**Figure 4.**
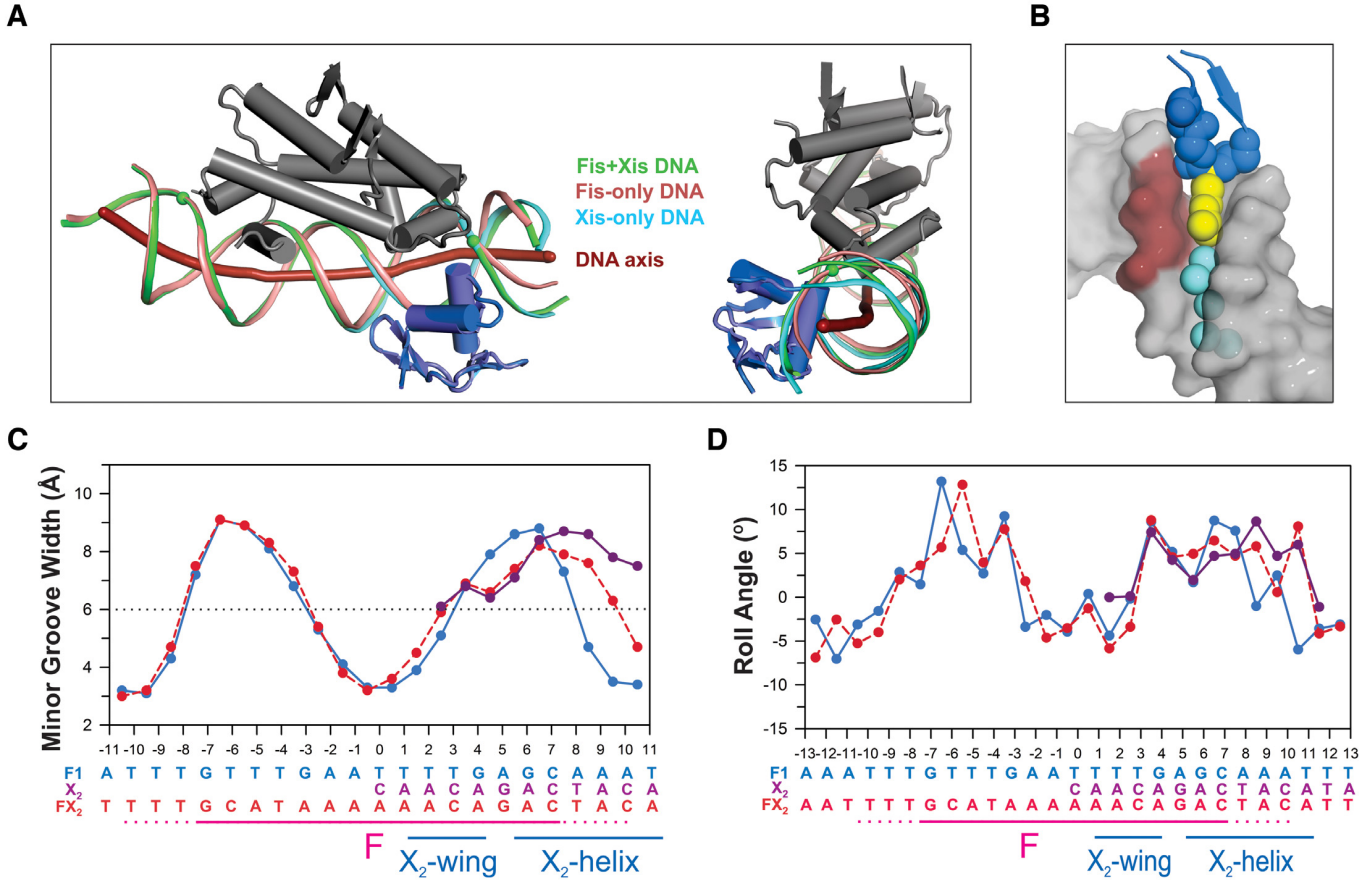
DNA conformational changes in the Fis+Xis complex. (**A**) DNA backbones and curvatures in Fis, Xis and Fis + Xis complexes. The Fis-F1 DNA structure (PDB code 3IV5) was aligned with the FX_2_ structure over the Fis dimers (rmsd = 0.28 Å between Fis dimer backbone atoms). Side and end-on views with the DNA axis of FX_2_ (thick red line, calculated by CURVES (27) are shown. The Fis dimer of FX_2_ is displayed and colored grey, the DNA backbone from FX_2_ is colored green with the spheres denoting the most distal phosphate contacts by Fis Asn73; Xis is colored blue. The DNA backbone of the Fis-F1 complex is colored salmon. Xis from the Xis-X2 structure (PDB code 1RH6, colored slate) was aligned with the Xis of FX_2_ (rmsd = 0.24 Å over backbone atoms); the Xis-only DNA backbone is colored cyan. The FX_2_ and Fis–DNA backbones closely align up to T9b, the most distal Fis-DNA contact. The FX_2_ DNA axis then bends towards Xis in a manner similar to the DNA in the Xis-only structure as evidenced by the close alignment of the green and cyan DNA strands proximal to Xis. (**B**) A view of the Xis wing inserted into the minor groove in the FX_2_ complex to highlight the close fit. Rendering is similar to Figure 2D with the CA dinucleotide at position 3-4 on the DNA top strand (chain C) colored dark red. (**C**) Plot of DNA minor groove widths (minus van der Waals radii) over the FX_2_ (red), Fis-F1 (blue), Xis-X2 (purple) complexes. Dashed line indicates the average minor groove width for B DNA. See Supplementary Figure S2 for minor groove plots of the FX_1-2_-1Xis and FX_1-2_-2Xis complexes. (**D**) Plots of DNA roll angles along the length of the DNA are shown for the FX_2_ (red), Fis-F1 (blue), Xis-X2 (purple) complexes.

The 27 bp DNA in the FX_2_ structure exhibits an overall curvature of 61° (calculated by CURVES), which is within the lower range of bending angles measured in X-ray structures of Fis bound to different DNA sequences (15,16). Xis binding, however, significantly alters the DNA trajectory over the X2 binding site within the F-X2 segment (Figure 4A). The DNA backbones from the canonical Fis-only (F1 sequence; (16)) and FX_2_ structures closely superimpose over the Fis binding interface to the most distal hydrogen bond, which is formed by Fis Asn73. The DNA backbone over the outer 4 bp of FX_2_ that is proximal to the Xis basic surface (Xis residues Arg14, Arg16, Arg23, Arg29 and Lys49) is then pulled up to 6 Å closer to Xis relative to the Fis-F1 complex, enabling hydrogen bonding by Xis Ser17, Thr20 and Trp24 to proximal phosphates. The resulting DNA structure over the X2 binding site in the FX_2_ complex closely follows the structures in Xis-X2 binary complexes (Figure 4A). The combined changes to the DNA trajectory by Fis and Xis binding are important for correctly positioning integrase arm and core binding sites on *attR* for assembly of the active synaptic complex (3,12).

Fis binding radically changes minor groove widths into a shape that corresponds to the minor groove architecture of Xis-only DNA complexes (Figure 4C). The minor groove on the DNA side opposite to where Fis inserts its recognition helices (bp 4–8) is expanded by almost 50% of average B DNA widths, even though major groove widths remain relatively constant (16). The minor groove then rapidly narrows to about half its canonical width at the center of the Fis interface. Minor groove widths in the Xis-only and Fis–Xis DNA complexes over the X2 binding site closely conform to those in Fis-only complexes, although when Xis is bound, there is an outward shift in the widening of the groove. This DNA backbone structure enables the peptide backbone of the Xis wing to insert into the minor groove towards the center of the Fis site such that Gly38 and Arg39 at the β-hairpin turn can protrude deep into the C–A dinucleotide step at bp 3-4 (Figure 4B). The result is a snug fit between the peptide backbone and the minor groove surface (surface complementarity score between the Xis wing motif and DNA in the FX_2_ structure is 0.826). The Arg39 side chain that emanates from the tip of the wing travels along the floor of the narrowing groove with its terminal amine engaging in multiple hydrogen bonding interactions with DNA atoms and a solvent molecule that begins an ordered hydration spine through the minor groove (also illustrated in Figure 6A, below). In addition to improving shape complementarity, the narrowing minor groove generates an increasingly negative electrostatic surface environment that favors insertion of the Arg39 side chain (43,44).

### DNA substitutions that alter Fis-induced changes in minor groove widths inhibit Fis–Xis cooperativity

**Figure 5.**
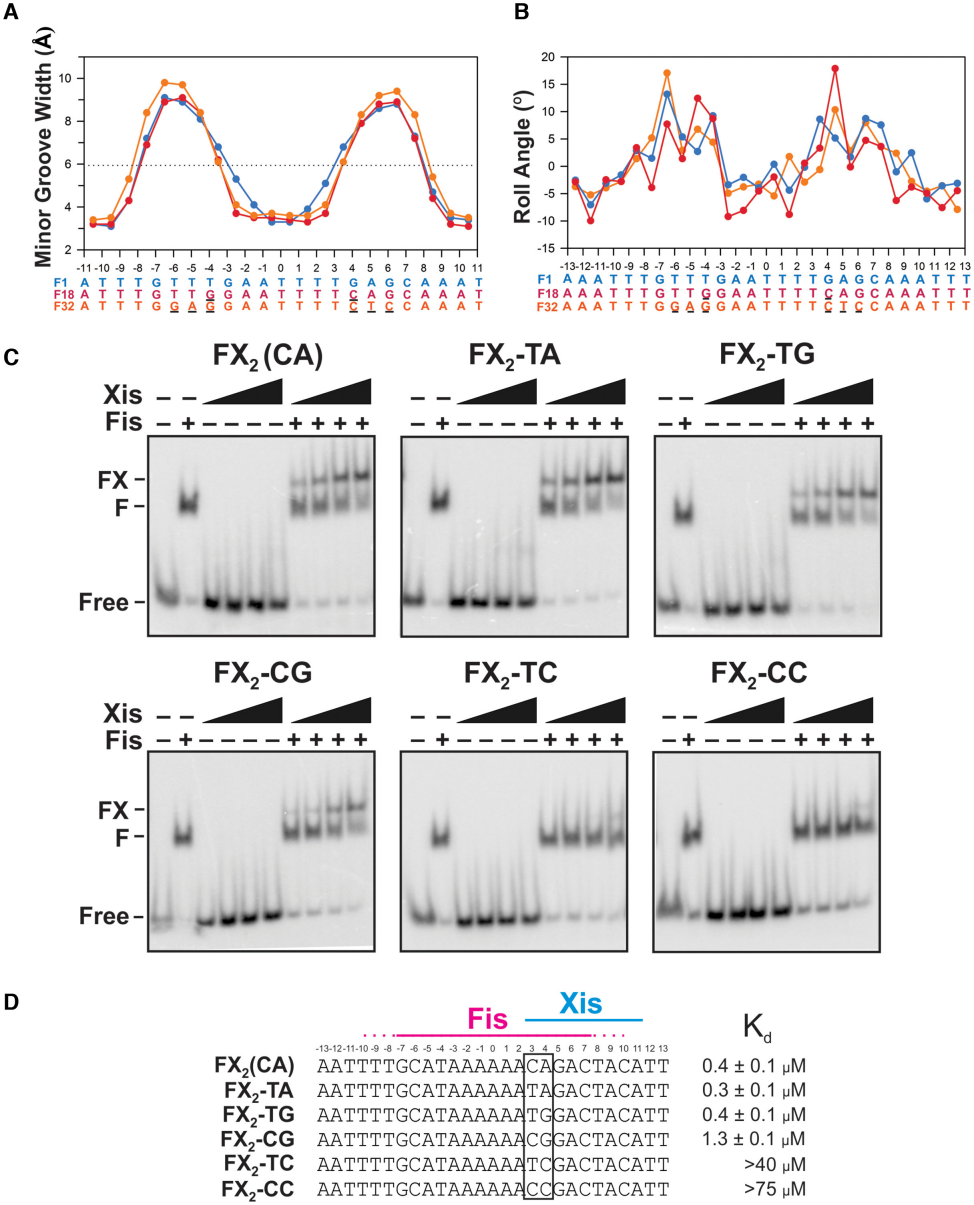
DNA base substitutions that are predicted to alter the conformation of Fis-bound DNA abrogate Xis recruitment by Fis. (**A**) Minor groove width plots for structures of Fis complexes with DNA containing sequence differences affecting the flexible pyrimidine-purine step within the Fis major groove interface. Blue plot is the F1 complex with a TG/CA dinucleotide step at ±(3–4) (PDB code 3IV5, (16)), red plot is the F18 complex with TG/CA at ±(4–5) (PDB code 3JRG, (16)) and orange plot is the F32 complex with no pyrimidine-purine step (pdb code 5E3O, (15)). (**B**) Roll angle plots of the same complexes in (A). (**C**) Gel mobility shift assays showing binding of Xis with and without Fis (4 nM) to 34 bp DNA duplexes containing the F-X2 binding sites with different dinucleotide steps at the 3–4 position. Free or Fis-bound DNA was incubated with increasing amounts of Xis (50, 150, 450, 1350 nM) for 20 min and subjected to PAGE. Xis does not bind without Fis to the short DNA fragment representing the native sequence (FX_2_ (CA)) under these conditions (8). (**D**) Sequences and apparent *K*_d_ values for Xis binding in the presence of Fis for each of the DNA variants at the 3–4 position tested. Means and standard deviations for the *K*_d_ values represent data from at least three replicate experiments.

In order to test the importance of Fis-induced DNA conformational changes in promoting Xis recruitment to *attR*, we evaluated Xis binding to Fis-DNA complexes that have been shown to exhibit altered minor groove shapes. When a flexible pyrimidine–purine (Y–R) base step at the ±(3–4) position is replaced by a Y–Y (R–R) step, the Fis-bound DNA exhibits a broader region of minor groove compression extending out from the center of the Fis binding site (Figure 5A, F18 and F32 Fis binding sequences (15,16)). In addition, the absence or displacement of the Y–R sequence step outwardly shifts the position of a Fis-induced kink in the DNA helical axis relative to the center of the Fis binding site (Figure 5B). We reasoned that if Fis-induced DNA structure promotes Xis binding, base substitutions that alter DNA shape in Fis-bound complexes over the Xis wing interface will compromise Xis recruitment.

**Figure 6.**
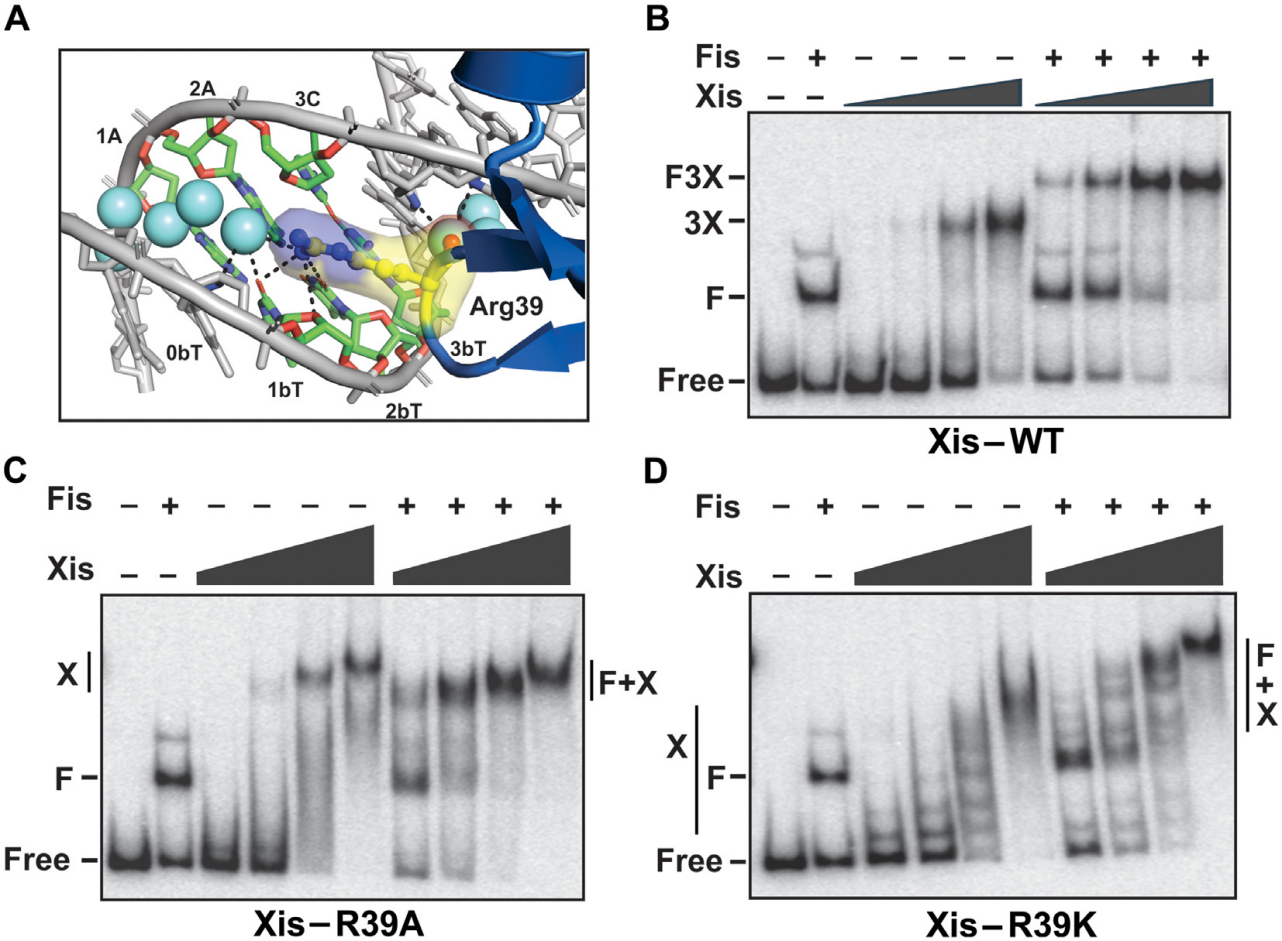
Role of Arginine 39 in Xis–DNA binding with and without Fis. (**A**) Arg39–DNA interactions within the minor groove in the FX_2_ structure. Dashes represent hydrogen bonds between Arg39, water (cyan spheres), and DNA. (B–D) Gel mobility shift assays of (**B**) Xis-wt and mutants (**C**) Arg39Ala and (**D**) Arg39Lys binding to *attR* with and without Fis. For Xis-wt, 25, 75, 225, and 675 nM were added; F denotes the Fis-bound DNA complex, 3X denotes 3 Xis protomers bound to X1–X1.5–X2 and F3X denotes the Fis + 3Xis complex. For Xis mutants, 1.5, 3.0, 6.0 and 12 μM were added; X denotes DNA complexes with multiple Xis protomers and F + X denotes complexes with Fis and multiple Xis protomers.

Fis–Xis cooperativity was evaluated on a 34 bp DNA fragment containing the *attR* F and X2 binding sites, similar to the duplex used for the FX_2_ structure. Xis-only binding to the short DNA probe is essentially undetectable, but Xis binds to the Fis-bound probe with a *K*_d_ ∼ 0.4 μM (Figure 5C and D) (see (8)). Xis binding to Fis–DNA complexes containing T–C or C–C at the +(3–4) step is severely reduced. Fis–Xis cooperativity remains, however, when the DNA contains the other Y–R step combinations at +(3–4), (T–A, T–G or C–G), suggesting that a flexible Y–R step, rather than the identity of the bases, is the critical determinant for Fis-dependent Xis binding at this position. We note that although the +(3–4) dinucleotide is not directly bonded by Xis, water-bridged interactions involving the Arg39 backbone carbonyl to minor groove atoms are present (see Figure 6A). However, similar hydrogen bonding to chemical groups on the minor groove floor would be expected to occur with different base pair identities.

Xis was absent or was bound in an improper manner at the X1 site in our X-ray structures employing the FX_1-2_ sequence, even though Xis bound properly to the X2 sequence (Figure 1D and E). This may be explained by the X1 sequence having an A–A (T–T) instead of a Y–R step at the 3–4 position of the Fis binding site. Thus, the native X1 sequence is incompatible with co-binding with Fis.

### Role of the Arg39 side chain in Xis and Fis–Xis binding

We evaluated the importance of the Arg39 side chain for Xis–DNA binding and recruitment by Fis. Binding by Xis mutants Arg39Ala or Arg39Lys to the 34 bp F-X2 probe was undetectable, even in the presence of Fis (not shown). Therefore, the 263 bp fragment containing the F–X2–X1.5–X1 region of *attR* was employed. Xis-wt protomers bind cooperatively to the three Xis binding sites, and under the conditions employed here, the three Xis protomers bind together with Fis at 2.5- to 3-fold higher affinity (Figure 6B). Xis-Arg39Ala forms cooperative but less stable complexes with *attR* at ∼15-fold lower affinity than Xis-wt (Figure 6C), confirming the importance of the arginine side chain for Xis binding. Significantly, Fis also enhances Arg39Ala binding by a factor of ∼2- to 2.5-fold. Interestingly, the electrophoretic mobility of the Xis-Arg39Ala complex formed with Fis is slightly faster than for the mutant Xis-only DNA complex. We verified that Fis is present in the Fis + Xis-Arg39Ala complex by the slower electrophoretic mobility generated using the higher MW derivative gfp-Fis (Supplementary Figure S3). The complexes formed by Xis-Arg39Ala both without and with Fis are less discrete and appear to increase in molecular weight with increasing Xis concentrations, implying formation of extended nucleoprotein filaments of Xis-Arg39Ala.

Xis–Arg39Lys binds *attR* DNA with about 10-fold lower affinity than Xis-wt, generating a stepwise increase in the number of complexes with added mutant Xis (Figure 6D). This non-cooperative binding profile is similar to Xis binding to non-*attR* probes (8), and indeed, Xis–Arg39Lys generated a similar stepwise increase in complexes on a non-specific DNA probe (data not shown). Moreover, Xis–Arg39Lys shows no evidence of cooperative binding with Fis as DNA complexes with Fis plus increasing Xis also exhibit stepwise shifts in apparent molecular weight (Figure 6D). We suggest that this altered DNA binding profile is caused by improper association of the wing with DNA, which results in non-specific and non-cooperative DNA binding. One model is that the mutant Lys39 side chain binds to a phosphate on the surface of DNA, in contrast to the native arginine side chain that associates with the floor of a narrowed minor groove. In this scenario, binding by Xis–Arg39Lys would be independent of minor groove widths, and Xis-Xis interactions promoted by acidic residues in the wing and basic residues on the opposite surface of an adjacently-bound Xis protomer (see (6)) would be disrupted because of the altered positioning of the wing.

We conclude that the Arg39 side chain is important for proper DNA binding by Xis and indirectly for Xis-Xis binding cooperativity, but does not directly contribute to Xis–Fis cooperativity.

### The symmetry-related Xis protomer over the X1 site of the FX_1-2_-2Xis complex resembles Xis bound to the X1.5 site in *attR*

**Figure 7.**
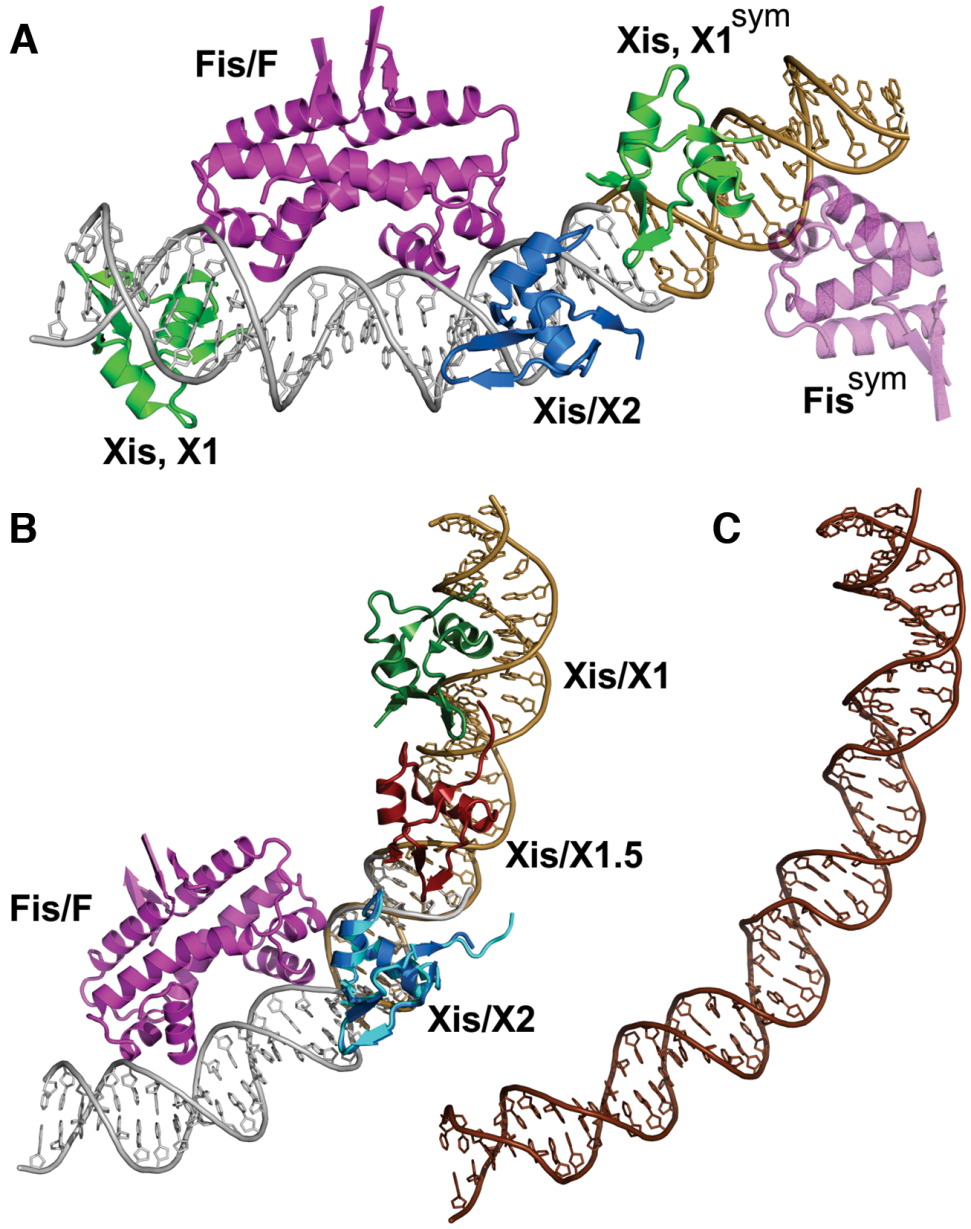
Model of the Fis–Xis region of λ *attR*. (**A**) The FX_1-2_-2Xis structure is shown together with a portion of its symmetry mate on the X2 side. The DNA of the symmetry mate is colored brown. Xis protomers bound over the X1 sequence are green with the wing of Xis, X1^sym^ oriented towards Xis bound at X2. Only half of the Fis dimer (transparent magenta) bound to the symmetry mate is displayed. A pseudo-continuous DNA helix extends from both DNA ends in the crystal lattice. (**B**) Model of the Fis–Xis complex over the F–X2–X1.5–X1 region. The Xis microfilament structure containing Xis bound to the X2–X1.5–X1 region (PDB code 2IEF, (6)) was aligned with the FX_2_ structure over the Xis protomers bound at X2 (Xis/X2 from FX_2_ colored blue and Xis/X2 from 2IEF colored cyan; rmsd over Xis backbone atoms = 0.42 Å). The DNA of 2IEF is brown. (**C**) The same DNA segment as shown in panel B (*attR* sequences 53–100) from the 11 Å cryoEM structure of the *attR/attL* intasome (PDB code 5J0N, (12)). The DNA is shown in the same orientation after alignment with the Fis–Xis model DNA in panel B. The cryoEM intasome structure did not include Fis.

The FX_1-2_-2Xis structure contains an Xis protomer bound over the region containing the X1 sequence but in reverse orientation (Figure 1E). The DNA in the crystal lattice forms a pseudo-continuous helix, and the Xis associated with X1 in the symmetry-related unit is abutted against the Xis at X2 with its wing protruding into the minor groove of the DNA duplex bound by X2 (Figure 7A). The configuration of the Xis^X2^-Xis^X1sym^ protomers resembles those bound to X1 and X1.5 in the X1–X1.5–X2 microfilament. In the X1–X1.5–X2 crystal structure, Xis protomers associate with each other predominantly through electrostatic interactions involving acidic residues on the wing (Asp37 and Glu40) and basic residues (most prominently Arg14, Arg16) on the adjacent Xis; however, the molecular interactions are not identical at the X2–X1.5 and X1.5–X1 interfaces due to differences in helical rotation between interacting Xis protomers (6). In the FX_1-2_-2Xis structure, the acidic residues on the symmetry-related Xis/X1 wing are also positioned close to Arg14 and Arg16 at X2, albeit their rotational relationship is not identical to either interface in the X1–X1.5–X2 crystal structure. The FX_1-2_-2Xis lattice thus approximates a Fis–Xis–Xis structure that contains an additional Xis positioned to interact with the Xis bound at X2.

We then combined the 2.6 Å resolution X2–X1.5–X1 structure (PDB code 2IEF) onto the FX_2_ structure by aligning the Xis protomers bound at X2 to generate a complete model of the ∼50 bp *attR* segment bound by Fis and Xis (Figure 7B). As expected, the overall trajectory of the DNA is very similar to the respective segment of the Xis-only bound *attR* arm in the 11 Å resolution cryo-EM structure of the λ excision complex (PDB code 5J0N, (12)) (Figure 7C). The excisive intasome in the cryo-EM structure was assembled without Fis so it is not unexpected that the DNA region over the F site exhibits greater deviation. In our model, Fis increases the curvature towards IHF bound to the H2 site. Importantly, when the DNA from our Fis–Xis model is aligned onto the respective segment of the cryo-EM structure, the Fis protein, including the extended N-terminal β-hairpin arms, does not interfere with other proteins or DNA segments within the intricately-wrapped intasome complex.

## DISCUSSION

Although phage λ excisive recombination can occur *in vitro* without Fis under high Xis concentrations, excision *in vivo* is largely dependent upon Fis. DNA excision products from an induced prophage were found to be nearly undetectable by Southern blotting (4) and decreased ∼100-fold by qPCR in *fis* mutant cells (8). Thompson *et al.* first showed that a high affinity Fis binding site overlaps the X2-Xis binding site in the λ *attR* region (19). This site is highly occupied in rapidly growing λ lysogens as measured in genome-wide chromatin immunoprecipation experiments (Y. Bernatavichute and R.C.J., unpublished data, see also (19)). Thus in growing lysogens, but not in quiescent cells when Fis levels are very low, Fis is poised to recruit Xis to *attR* to initiate excision when Xis and Int expression are induced upon DNA damage (4,5,19,45). *In vitro*, Xis by itself binds DNA with low affinity and in a largely non-specific manner (8,10). Even at *attR*, which contains three adjacent Xis binding sites in helical phase with each other, Xis binds with 100-fold lower affinity than Fis in the presence of excess competitor DNA (18). On short DNA substrates containing only the F and X2 sites, Xis binding is essentially dependent on Fis (Figure 5C and (8)). Fis promotes Xis loading to the X1–X1.5–X2 segment of *attR* with up to 4-fold higher affinity (8,18,19). We show here that recruitment of Xis to X2 by Fis is not through direct protein–protein interactions, but rather indirectly through DNA conformation.

The Xis (X2) and Fis (F) binding sites in *attR* overlap, though the bound proteins are rotationally displaced about 70° from each other on DNA in the co-complex. Our three crystal structures of Fis and Xis bound to the F-X2 DNA segment reveal little (70 Å^2^ in the FX_2_ structure) or no (both FX_1-2_ structures) shared surface between the Xis and Fis proteins. A curtain of ordered solvent molecules separates amino acid residues of the Fis and Xis DNA recognition helices that are inserted antiparallel with respect to each other within the major groove. The closest approach in the FX_2_ structure is between the polar end of the Xis Gln6 side chain from helix A and the Fis Leu79 side chain methyl, which emanates from its positioning helix C (Figure 2D). However, in the FX_1-2_-2Xis structure, the Gln6 side chain is oriented to contact a DNA phosphate.

Previous studies have shown that Fis Arg85 and Xis Glu19 make the most important base contacts in their respective individual DNA complexes (16,17). In the co-complex these residues hydrogen bond to the same G/C base pair, and are indirectly hydrogen bonded through two intervening water molecules (Figure 2B and C). Nevertheless, Xis-Glu19Ala is still recruited to *attR* by Fis, even under conditions where the mutant exhibited undetectable binding to *attR* on its own (Figure 2E). Gardner and coworkers also reported that this mutant exhibited intermediate levels of excision *in vivo* when Fis was present (42). We conclude that direct or indirect interactions between these residues cannot be essential for cooperative binding.

The lack of protein–protein interactions between Fis and Xis led us to consider a mechanism by which binding cooperativity is mediated through DNA structure. Fis binding narrows the minor groove to about half its normal width at the center of its binding site and expands the minor groove about 50% over average B DNA widths, peaking 6 bp from the center (Figure 4C). The Fis + Xis structures reveal how the Fis-induced changes in minor groove width are tailored for binding by the Xis wing. The β-hairpin turn at the tip of wing snugly inserts into an expanded minor groove on the opposing face of the DNA helix from Fis to position the Arg39 side chain along the floor of an increasingly narrowing groove. Fis thus conforms the minor groove into a shape and surface charge potential (44,46) that is optimized for Xis binding.

The properties of mutations in both the DNA sequence and at Xis Arg39 provide experimental support that proper insertion of the wing peptide backbone is of primary importance for Fis–Xis cooperativity, with the resulting interactions between Arg39 and the minor groove floor strongly contributing to Xis-DNA affinity. Previous studies have documented that DNA mutations which eliminate or shift the position of the Y-R base pair step, which is optimally located at the (±)3–4 steps in high affinity Fis binding sites, change the minor groove shape in Fis-bound complexes (15,16). In these complexes, the minor groove is too narrow for insertion of the Xis wing, and indeed, we find that Fis–Xis cooperativity is lost with mutations that eliminate a Y–R step at the 3–4 position (Figure 5). The Y–R step at the 3-4 position of Fis, Xis and Fis–Xis complexes all exhibit a modest kink (7.5–10°, Figure 4D), which may also contribute to DNA shape-based cooperative binding.

Without an arginine side chain at residue 39, Xis–DNA binding affinity is severely decreased; however, Fis is still able to recruit Xis-Arg39Ala to *attR*. It is noteworthy that a lysine will not functionally substitute for an arginine at residue 39; rather, Xis–Arg39Lys exhibits sequence-independent non-cooperative binding with or without Fis. Insertion of arginines into narrow A/T-rich minor grooves are a common feature within protein-DNA complexes. Arginines are believed to be preferred over lysines within the minor groove because of the lower energetic cost of removing a charged arginine as compared to lysine from water and the greater strength of arginine cation-π interactions relative to lysine in biological environments (46,47). The binding properties of Xis–Arg39Lys suggests that the mutant is not binding DNA in the correct manner and highlights the importance of a properly bound wing for Xis–Xis cooperativity, which is driven primarily by electrostatic interactions between acidic residues on the wing and basic residues on the body of the partner Xis (6). Sequence-independent non-cooperative binding profiles similar to Xis–Arg39Lys (but not Arg39Ala) are a hallmark of mutants containing changes that eliminate basic or acidic residues in the Xis-Xis interface (6).

Fis also strongly bends DNA, including small (∼–15°) out-of-plane bends at each DNA end (15,16). Xis binding to the X2 site induces about 25° of overall curvature originating from its major groove binding interface (6,17). Because the Fis- and Xis-induced curvatures over the X2 segment of the F-X2 locus are largely co-planar (Figure 4A), Fis-induced bending further conforms the DNA into an optimal shape for Xis binding. In support of the role of Fis-induced bending, Fis mutants Thr75Ala and Asn73Ala have a measureable effect on Xis recruitment (8). These residues are not close to Xis, but their side chains make DNA backbone contacts at the outer edge of the Fis-DNA binding interface and thus are likely to influence the DNA trajectory over X2. We note that the opposite (H2 proximal) side of the *attR* F binding site has an A-tract over the sequence that would correspond to the position where the Xis recognition helix would insert (see Figure 2C), and thus this sequence would probably discourage Xis binding.

In summary, our structures of Fis-, Xis- and Fis–Xis DNA complexes, along with binding properties of DNA and protein mutants strongly suggest Fis recruits Xis to *attR* through a DNA allostery mechanism. Fis binding both molds the minor groove to optimize insertion of the Xis wing backbone and Arg39 side chain within the groove and shapes the major groove to promote Xis contacts to the DNA backbone. These DNA structural changes induced by Fis combine to conform a lower energy binding target for Xis.

We emphasize two features of DNA-mediated cooperativity in the Fis–Xis complex that may be generally applicable to other cooperative binding complexes: critical DNA conformational changes can be relatively small and must be highly localized. For example, the minor groove is only modestly expanded where the Xis wing backbone abuts the DNA backbone, but the geometry of this expanded groove precisely fits the Xis wing structure at the site of insertion, as evidenced by experiments showing that a 1–2 bp shift abrogates cooperative binding (Figure 5). Likewise, the relatively small amount of Fis-induced curvature introduced into the major groove binding interface of Xis must be appropriately helically-phased such that modest additional bending in the proper rotational phase can enable hydrogen bonding by Xis residues (e.g. Ser17, Thr20 and Trp24) to the DNA backbone.

The eukaryotic chromatin landscape contains dense arrays of different DNA binding proteins within nucleosome-free regions (48,49). As noted above for the β-interferon enhancer (1), protein–protein interactions facilitating formation of these arrays are not always evident. A recent high throughput study identified hundreds of examples where pairs of eukaryotic transcription factors bound to abutting or overlapping DNA sequences in a random DNA library (50). In most cases, little, if any, direct protein–protein interactions were implicated, implying that DNA-mediated mechanisms contributed to co-complex formation. Indeed, a crystal structure of one pair (two MEIS1 homeodomains) bound to opposite sides of an overlapping sequence confirmed the absence of any protein–protein interactions. Even when protein–protein interactions dominate, there can be compelling evidence for cooperative binding through DNA conformation. For example, the POU-specific domain and the POU homeodomain bind cooperatively to the Oct-1 site even when the peptide segment that normally covalently links these DNA binding domains is removed (51). Hox-Exd/Pbx co-complexes are stabilized by noncovalent interactions between the N-terminal end of the Hox protein and a cognate binding pocket on Exd. Although removal of N-terminal docking peptide of the Hox protein Ubx reduces Exd binding 100-fold, Exd binding was found to be still 10-fold greater than without the mutant Ubx (52). Remarkably, a sequence-specific polyamide in place of Ubx also enhanced Exd binding by a similar amount, and it was proposed that the modest expansion in minor groove width by binding of the polyamide was responsible for the increased Exd binding within the adjacent major groove. This stimulation required precise positioning of the polyamide, similar to the precise minor groove conformation required for recruitment of Xis by Fis. Another potential example involving Fis occurs during activation of transcription whereby Fis stabilizes RNA polymerase binding through the sigma subunit, but effects of mutations within the proposed Fis-sigma interface are modest (53). These and other examples (see (2,43,54,55)) suggest that cooperative protein-DNA binding mediated solely or in part through relatively small DNA conformational changes may be common where overlapping or closely juxtaposed binding sites are involved.

## DATA AVAILABILITY

Atomic coordinates and structure factors for the reported crystal structures are available from the Protein Data bank under accession numbers 6P0S, 6P0T and 6P0U.

## Supplementary Material

gkz642_Supplemental_File

## References

[1] Panne D., Maniatis T., Harrison S.C. An atomic model of the interferon-beta enhanceosome. Cell. 2007; 129:1111–1123. 17574024 10.1016/j.cell.2007.05.019 PMC2020837

[2] Kim S., Brostromer E., Xing D., Jin J., Chong S., Ge H., Wang S., Gu C., Yang L., Gao Y.Q. et al. Probing allostery through DNA. Science. 2013; 339:816–819. 23413354 10.1126/science.1229223 PMC3586787

[3] Landy A. The lambda integrase site-specific recombination pathway. Microb. Spectr. 2015; 3:doi:10.1128/microbiolspec.MDNA3-0051-2014. 10.1128/microbiolspec.MDNA3-0051-2014 PMC5710010 26104711

[4] Ball C.A., Johnson R.C. Efficient excision of phage lambda from the *Escherichia coli* chromosome requires the Fis protein. J. Bacteriol. 1991; 173:4027–4031. 1829453 10.1128/jb.173.13.4027-4031.1991 PMC208050

[5] Thompson J.F., Landy A. Berg D, Howe M Mobile DNA. 1989; Washington ASM Press 1–22.

[6] Abbani M.A., Papagiannis C.V., Sam M.D., Cascio D., Johnson R.C., Clubb R.T. Structure of the cooperative Xis-DNA complex reveals a micronucleoprotein filament that regulates phage lambda intasome assembly. Proc. Natl. Acad. Sci. U.S.A. 2007; 104:2109–2114. 17287355 10.1073/pnas.0607820104 PMC1893000

[7] Sun X., Mierke D.F., Biswas T., Lee S.Y., Landy A., Radman-Livaja M. Architecture of the 99 bp DNA-six-protein regulatory complex of the lambda *att* site. Mol. Cell. 2006; 24:569–580. 17114059 10.1016/j.molcel.2006.10.006 PMC1866956

[8] Papagiannis C.V., Sam M.D., Abbani M.A., Yoo D., Cascio D., Clubb R.T., Johnson R.C. Fis targets assembly of the Xis nucleoprotein filament to promote excisive recombination by phage lambda. J. Mol. Biol. 2007; 367:328–343. 17275024 10.1016/j.jmb.2006.12.071 PMC1852488

[9] Warren D., Sam M.D., Manley K., Sarkar D., Lee S.Y., Abbani M., Wojciak J.M., Clubb R.T., Landy A. Identification of the lambda integrase surface that interacts with Xis reveals a residue that is also critical for Int dimer formation. Proc. Natl. Acad. Sci. U.S.A. 2003; 100:8176–8181. 12832614 10.1073/pnas.1033041100 PMC166202

[10] Bushman W., Yin S., Thio L.L., Landy A. Determinants of directionality in lambda site-specific recombination. Cell. 1984; 39:699–706. 6239693 10.1016/0092-8674(84)90477-x

[11] Sarkar D., Azaro M.A., Aihara H., Papagiannis C.V., Tirumalai R., Nunes-Duby S.E., Johnson R.C., Ellenberger T., Landy A. Differential affinity and cooperativity functions of the amino-terminal 70 residues of lambda integrase. J. Mol. Biol. 2002; 324:775–789. 12460577 10.1016/s0022-2836(02)01199-3

[12] Laxmikanthan G., Xu C., Brilot A.F., Warren D., Steele L., Seah N., Tong W., Grigorieff N., Landy A., Van Duyne G.D. Structure of a Holliday junction complex reveals mechanisms governing a highly regulated DNA transaction. Elife. 2016; 5:e14313. 27223329 10.7554/eLife.14313 PMC4880445

[13] Finkel S.E., Johnson R.C. The Fis protein: it's not just for DNA inversion anymore. Mol. Microbiol. 1992; 6:3257–3265. 1484481 10.1111/j.1365-2958.1992.tb02193.x

[14] Hancock S.P., Ghane T., Cascio D., Rohs R., Di Felice R., Johnson R.C. Control of DNA minor groove width and Fis protein binding by the purine 2-amino group. Nucleic Acids Res. 2013; 41:6750–6760. 23661683 10.1093/nar/gkt357 PMC3711457

[15] Hancock S.P., Stella S., Cascio D., Johnson R.C. DNA sequence determinants controlling affinity, stability and shape of DNA complexes bound by the nucleoid protein Fis. PLoS One. 2016; 11:e0150189. 26959646 10.1371/journal.pone.0150189 PMC4784862

[16] Stella S., Cascio D., Johnson R.C. The shape of the DNA minor groove directs binding by the DNA-bending protein Fis. Genes Dev. 2010; 24:814–826. 20395367 10.1101/gad.1900610 PMC2854395

[17] Sam M.D., Cascio D., Johnson R.C., Clubb R.T. Crystal structure of the excisionase-DNA complex from bacteriophage lambda. J. Mol. Biol. 2004; 338:229–240. 15066428 10.1016/j.jmb.2004.02.053

[18] Sam M.D., Papagiannis C.V., Connolly K.M., Corselli L., Iwahara J., Lee J., Phillips M., Wojciak J.M., Johnson R.C., Clubb R.T. Regulation of directionality in bacteriophage lambda site-specific recombination: structure of the Xis protein. J. Mol. Biol. 2002; 324:791–805. 12460578 10.1016/s0022-2836(02)01150-6

[19] Thompson J.F., Moitoso de Vargas L., Koch C., Kahmann R., Landy A. Cellular factors couple recombination with growth phase: characterization of a new component in the lambda site-specific recombination pathway. Cell. 1987; 50:901–908. 2957063 10.1016/0092-8674(87)90516-2

[20] Numrych T.E., Gumport R.I., Gardner J.F. Characterization of the bacteriophage lambda excisionase (Xis) protein: the C-terminus is required for Xis-integrase cooperativity but not for DNA binding. EMBO J. 1992; 11:3797–3806. 1396573 10.1002/j.1460-2075.1992.tb05465.x PMC556840

[21] Kabsch W. XDS. Acta Crystallogr. D. Biol. Crystallogr. 2010; 66:125–132. 20124692 10.1107/S0907444909047337 PMC2815665

[22] McCoy A.J., Grosse-Kunstleve R.W., Adams P.D., Winn M.D., Storoni L.C., Read R.J. Phaser crystallographic software. J. Appl. Crystallogr. 2007; 40:658–674. 19461840 10.1107/S0021889807021206 PMC2483472

[23] Emsley P., Cowtan K. Coot: model-building tools for molecular graphics. Acta Crystallogr. D. Biol. Crystallogr. 2004; 60:2126–2132. 15572765 10.1107/S0907444904019158

[24] Adams P.D., Grosse-Kunstleve R.W., Hung L.W., Ioerger T.R., McCoy A.J., Moriarty N.W., Read R.J., Sacchettini J.C., Sauter N.K., Terwilliger T.C. PHENIX: building new software for automated crystallographic structure determination. Acta Crystallogr. D. Biol. Crystallogr. 2002; 58:1948–1954. 12393927 10.1107/s0907444902016657

[25] Smart O.S., Womack T.O., Flensburg C., Keller P., Paciorek W., Sharff A., Vonrhein C., Bricogne G. Exploiting structure similarity in refinement: automated NCS and target-structure restraints in BUSTER. Acta Crystallogr. D. Biol. Crystallogr. 2012; 68:368–380. 22505257 10.1107/S0907444911056058 PMC3322596

[26] Lu X.J., Olson W.K. 3DNA: a versatile, integrated software system for the analysis, rebuilding and visualization of three-dimensional nucleic-acid structures. Nat. Protoc. 2008; 3:1213–1227. 18600227 10.1038/nprot.2008.104 PMC3065354

[27] Lavery R., Sklenar H. The definition of generalized helicoidal parameters and of axis curvature for irregular nucleic acids. J. Biomol. Struct. Dyn. 1988; 6:63–91. 2482765 10.1080/07391102.1988.10506483

[28] Lawrence M.C., Colman P.M. Shape complementarity at protein/protein interfaces. J. Mol. Biol. 1993; 234:946–950. 8263940 10.1006/jmbi.1993.1648

[29] Lee B., Richards F.M. The interpretation of protein structures: estimation of static accessibility. J. Mol. Biol. 1971; 55:379–400. 5551392 10.1016/0022-2836(71)90324-x

[30] Winn M.D., Ballard C.C., Cowtan K.D., Dodson E.J., Emsley P., Evans P.R., Keegan R.M., Krissinel E.B., Leslie A.G., McCoy A. et al. Overview of the CCP4 suite and current developments. Acta Crystallogr. D. Biol. Crystallogr. 2011; 67:235–242. 21460441 10.1107/S0907444910045749 PMC3069738

[31] Chiu T.K., Sohn C., Dickerson R.E., Johnson R.C. Testing water-mediated DNA recognition by the Hin recombinase. EMBO J. 2002; 21:801–814. 11847127 10.1093/emboj/21.4.801 PMC125850

[32] Kostrewa D., Granzin J., Koch C., Choe H.W., Raghunathan S., Wolf W., Labahn J., Kahmann R., Saenger W. Three-dimensional structure of the *E. coli* DNA-binding protein FIS. Nature. 1991; 349:178–180. 1986310 10.1038/349178a0

[33] Yuan H.S., Finkel S.E., Feng J.A., Kaczor-Grzeskowiak M., Johnson R.C., Dickerson R.E. The molecular structure of wild-type and a mutant Fis protein: relationship between mutational changes and recombinational enhancer function or DNA binding. Proc. Natl. Acad. Sci. U.S.A. 1991; 88:9558–9562. 1946369 10.1073/pnas.88.21.9558 PMC52757

[34] Cho B.K., Knight E.M., Barrett C.L., Palsson B.O. Genome-wide analysis of Fis binding in *Escherichia coli* indicates a causative role for A-/AT-tracts. Genome Res. 2008; 18:900–910. 18340041 10.1101/gr.070276.107 PMC2413157

[35] Hengen P.N., Bartram S.L., Stewart L.E., Schneider T.D. Information analysis of Fis binding sites. Nucleic Acids Res. 1997; 25:4994–5002. 9396807 10.1093/nar/25.24.4994 PMC147151

[36] Kahramanoglou C., Seshasayee A.S., Prieto A.I., Ibberson D., Schmidt S., Zimmermann J., Benes V., Fraser G.M., Luscombe N.M. Direct and indirect effects of H-NS and Fis on global gene expression control in *Escherichia coli*. Nucleic Acids Res. 2011; 39:2073–2091. 21097887 10.1093/nar/gkq934 PMC3064808

[37] Shao Y., Feldman-Cohen L.S., Osuna R. Functional characterization of the *Escherichia coli* Fis-DNA binding sequence. J. Mol. Biol. 2008; 376:771–785. 18178221 10.1016/j.jmb.2007.11.101 PMC2292415

[38] Pan C.Q., Finkel S.E., Cramton S.E., Feng J.A., Sigman D.S., Johnson R.C. Variable structures of Fis-DNA complexes determined by flanking DNA-protein contacts. J. Mol. Biol. 1996; 264:675–695. 8980678 10.1006/jmbi.1996.0669

[39] Feldman-Cohen L.S., Shao Y., Meinhold D., Miller C., Colon W., Osuna R. Common and variable contributions of Fis residues to high-affinity binding at different DNA sequences. J. Bacteriol. 2006; 188:2081–2095. 16513738 10.1128/JB.188.6.2081-2095.2006 PMC1428148

[40] Osuna R., Finkel S.E., Johnson R.C. Identification of two functional regions in Fis: the N-terminus is required to promote Hin-mediated DNA inversion but not lambda excision. EMBO J. 1991; 10:1593–1603. 1851089 10.1002/j.1460-2075.1991.tb07680.x PMC452825

[41] Koch C., Ninnemann O., Fuss H., Kahmann R. The N-terminal part of the *E. coli* DNA binding protein FIS is essential for stimulating site-specific DNA inversion but is not required for specific DNA binding. Nucleic Acids Res. 1991; 19:5915–5922. 1834996 10.1093/nar/19.21.5915 PMC329047

[42] Cho E.H., Alcaraz R. Jr, Gumport R.I., Gardner J.F. Characterization of bacteriophage lambda excisionase mutants defective in DNA binding. J. Bacteriol. 2000; 182:5807–5812. 11004181 10.1128/jb.182.20.5807-5812.2000 PMC94704

[43] Abe N., Dror I., Yang L., Slattery M., Zhou T., Bussemaker H.J., Rohs R., Mann R.S. Deconvolving the recognition of DNA shape from sequence. Cell. 2015; 161:307–318. 25843630 10.1016/j.cell.2015.02.008 PMC4422406

[44] Chiu T.P., Rao S., Mann R.S., Honig B., Rohs R. Genome-wide prediction of minor-groove electrostatic potential enables biophysical modeling of protein-DNA binding. Nucleic Acids Res. 2017; 45:12565–12576. 29040720 10.1093/nar/gkx915 PMC5716191

[45] Ball C.A., Osuna R., Ferguson K.C., Johnson R.C. Dramatic changes in Fis levels upon nutrient upshift in *Escherichia coli*. J. Bacteriol. 1992; 174:8043–8056. 1459953 10.1128/jb.174.24.8043-8056.1992 PMC207543

[46] Rohs R., West S.M., Sosinsky A., Liu P., Mann R.S., Honig B. The role of DNA shape in protein-DNA recognition. Nature. 2009; 461:1248–1253. 19865164 10.1038/nature08473 PMC2793086

[47] Kumar K., Woo S.M., Siu T., Cortopassi W.A., Duarte F., Paton R.S. Cation-pi interactions in protein-ligand binding: theory and data-mining reveal different roles for lysine and arginine. Chem. Sci. 2018; 9:2655–2665. 29719674 10.1039/c7sc04905f PMC5903419

[48] Moorman C., Sun L.V., Wang J., de Wit E., Talhout W., Ward L.D., Greil F., Lu X.J., White K.P., Bussemaker H.J. et al. Hotspots of transcription factor colocalization in the genome of *Drosophila melanogaster*. Proc. Natl. Acad. Sci. U.S.A. 2006; 103:12027–12032. 16880385 10.1073/pnas.0605003103 PMC1567692

[49] Gerstein M.B., Kundaje A., Hariharan M., Landt S.G., Yan K.K., Cheng C., Mu X.J., Khurana E., Rozowsky J., Alexander R. et al. Architecture of the human regulatory network derived from ENCODE data. Nature. 2012; 489:91–100. 22955619 10.1038/nature11245 PMC4154057

[50] Jolma A., Yin Y., Nitta K.R., Dave K., Popov A., Taipale M., Enge M., Kivioja T., Morgunova E., Taipale J. DNA-dependent formation of transcription factor pairs alters their binding specificity. Nature. 2015; 527:384–388. 26550823 10.1038/nature15518

[51] Klemm J.D., Pabo C.O. Oct-1 POU domain-DNA interactions: cooperative binding of isolated subdomains and effects of covalent linkage. Genes Dev. 1996; 10:27–36. 8557192 10.1101/gad.10.1.27

[52] Moretti R., Donato L.J., Brezinski M.L., Stafford R.L., Hoff H., Thorson J.S., Dervan P.B., Ansari A.Z. Targeted chemical wedges reveal the role of allosteric DNA modulation in protein-DNA assembly. ACS Chem. Biol. 2008; 3:220–229. 18422304 10.1021/cb700258r PMC3060767

[53] Typas A., Stella S., Johnson R.C., Hengge R. The -35 sequence location and the Fis-sigma factor interface determine sigmas selectivity of the *proP* (P2) promoter in *Escherichia coli*. Mol. Microbiol. 2007; 63:780–796. 17302803 10.1111/j.1365-2958.2006.05560.x

[54] Morgunova E., Taipale J. Structural perspective of cooperative transcription factor binding. Curr. Opin. Struct. Biol. 2017; 47:1–8. 28349863 10.1016/j.sbi.2017.03.006

[55] Ogata K., Sato K., Tahirov T.H. Eukaryotic transcriptional regulatory complexes: cooperativity from near and afar. Curr. Opin. Struct. Biol. 2003; 13:40–48. 12581658 10.1016/s0959-440x(03)00012-5

