# Supplementary material for: Cooperative DNA binding by proteins through DNA shape complementarity
Source: Nucleic Acids Res. 2019 Jul 25;47(16):8874–87. doi: 10.1093/nar/gkz642 (PMC7145599; doi:10.1093/nar/gkz642)
Supplement: gkz642_Supplemental_File [file gkz642_supplemental_file.pdf]

Supplementary figures for:

Cooperative DNA Binding by Proteins through DNA Shape Complementarity

Stephen P. Hancock, Duilio Cascio, and Reid C. Johnson

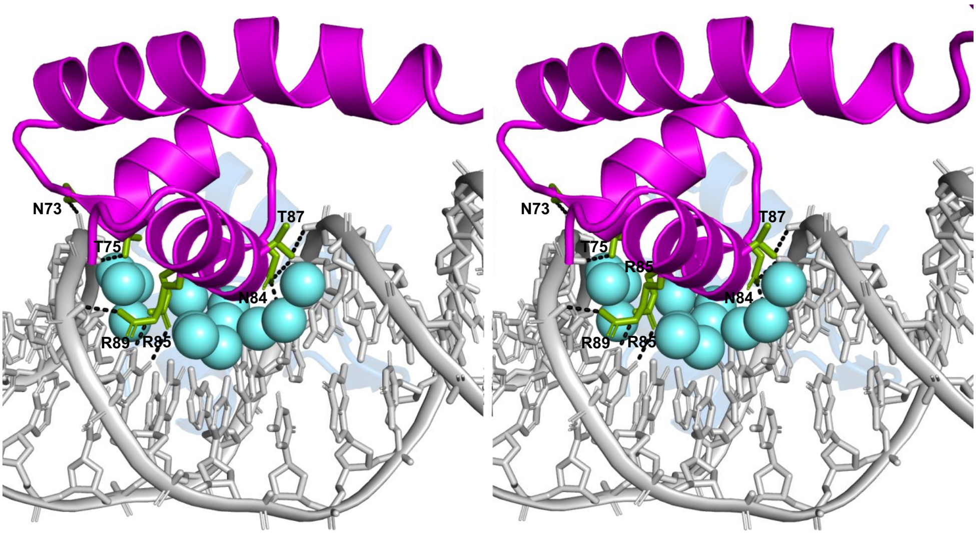

Figure S1. Wall-eyed stereo pair of the solvated Fis-DNA interface in the FX_2 structure. The Fis helix-turn-helix motif is in magenta and water molecules within the Fis-DNA interface are shown as cyan spheres. The light blue ribbon behind Fis is the Xis protomer bound to X2. Selected Fis side chains (green) making direct hydrogen bonds (black dashed lines) with DNA atoms are shown.

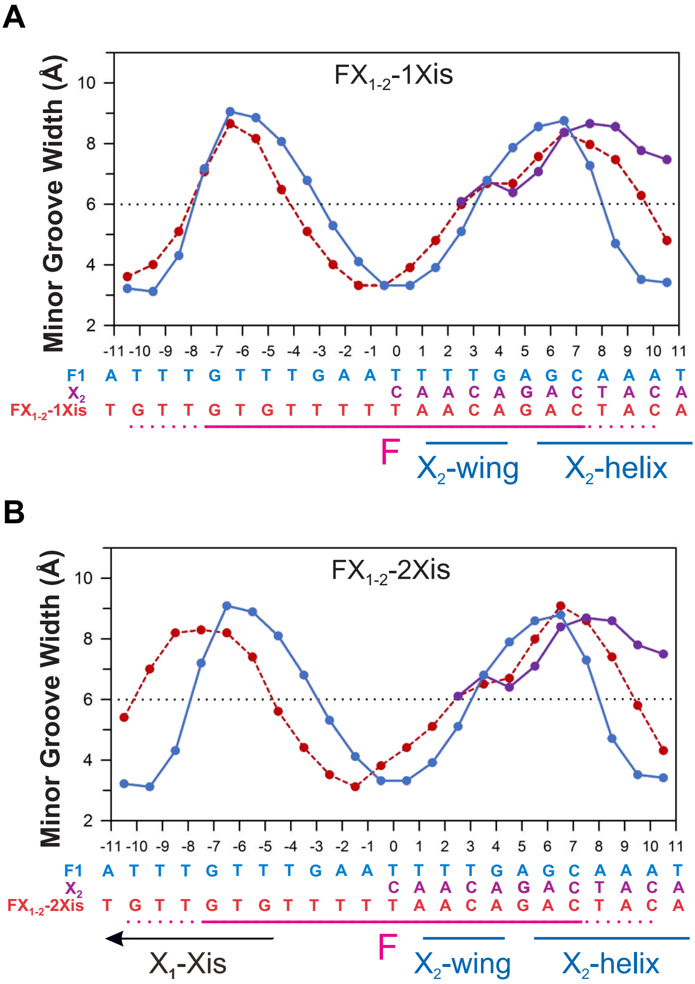

Figure S2. Minor groove width plots of: (**A**) $FX_{1\text{-}2}$-1Xis and (**B**) $FX_{1\text{-}2}$-2Xis complexes. For comparison, the minor groove widths of DNA complexes containing only Fis (F1 complex, PDB code 3IV5) or Xis (1RH6) are also shown; each plot is color coordinated with the sequence. The $FX_{1\text{-}2}$-2Xis complex contains an additional Xis non-specifically bound over the X1 region in reverse orientation, which is probably responsible for the shift in minor groove widths relative to Fis on the left end.

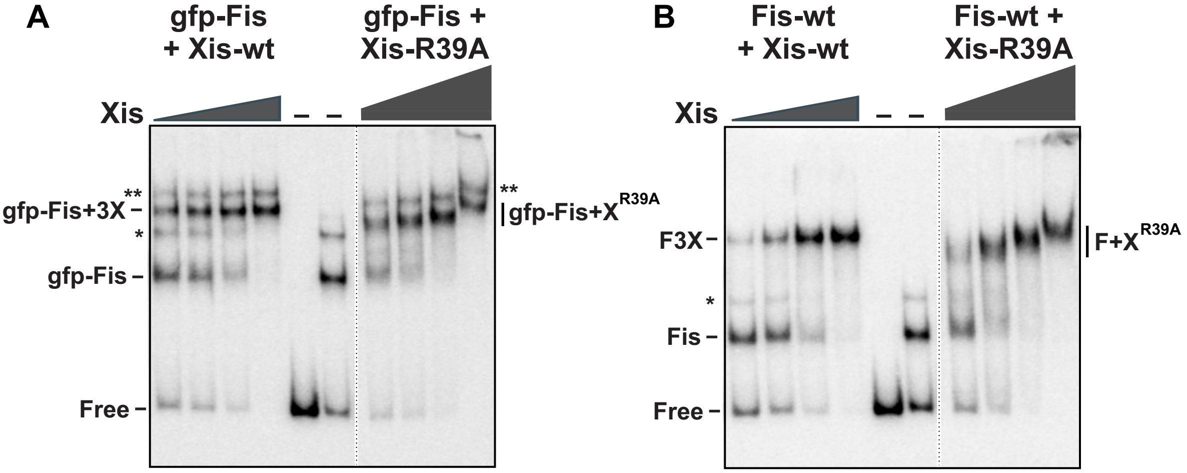

Figure S3. Complexes formed with Fis and Xis-Arg39Ala contain Fis. (**A**) Complexes formed by gfp-Fis and Xis-wt or Xis-Arg39Ala. (**B**) Complexes formed by Fis-wt and Xis-wt or Xis-Arg39Ala. As in Figure 6, Xis-wt was added at 25, 75, 225, and 675 nM; Xis-Arg39Ala was added at 1.5, 3.0, 6.0, and 12 µM; gfp-Fis or Fis were added at about 4 nM. The mobilities of the complexes are labeled; (*) designates the mobility of a minor complex containing a second gfp-Fis or Fis-wt dimer, and (**) designates the two Fis dimer complex with Xis.

The experiments in panels A and B were performed in parallel, but electrophoresis was in separate gels. The slower mobility of the Xis complexes formed with gfp-Fis (dimer MW = 76.5 kDa) relative to Fis-wt (dimer MW = 22.5 kDa) confirms that Fis is present in the complex.

gfp-Fis, containing eGFP inserted near the N-terminus of Fis, is described in Graham et al. (2011) *Nucleic Acids Res*. **39**, 2249-2259.
